# Discovery of Novel Biomarkers for Alzheimer's Disease from Blood

**DOI:** 10.1155/2016/4250480

**Published:** 2016-05-29

**Authors:** Jintao Long, Genhua Pan, Emmanuel Ifeachor, Robert Belshaw, Xinzhong Li

**Affiliations:** ^1^School of Medicine, Plymouth University Peninsula Schools of Medicine and Dentistry (PUPSMD), Drake Circus, Plymouth PL4 8AA, UK; ^2^School of Computing, Electronics and Mathematics, Plymouth University, Drake Circus, Plymouth PL4 8AA, UK; ^3^School of Biomedical & Healthcare Sciences, Plymouth University Peninsula Schools of Medicine and Dentistry (PUPSMD), Drake Circus, Plymouth PL4 8AA, UK

## Abstract

Blood-based biomarkers for Alzheimer's disease would be very valuable because blood is a more accessible biofluid and is suitable for repeated sampling. However, currently there are no robust and reliable blood-based biomarkers for practical diagnosis. In this study we used a knowledge-based protein feature pool and two novel support vector machine embedded feature selection methods to find panels consisting of two and three biomarkers. We validated these biomarker sets using another serum cohort and an RNA profile cohort from the brain. Our panels included the proteins ECH1, NHLRC2, HOXB7, FN1, ERBB2, and SLC6A13 and demonstrated promising sensitivity (>87%), specificity (>91%), and accuracy (>89%).

## 1. Introduction

There were an estimated 46.8 million Alzheimer's disease (AD) sufferers worldwide in 2015, and it is predicted that 1 in 85 people will be affected by 2050 [1]. Although a number of genetic and cerebrospinal fluid (CSF) biomarkers have been discovered in recent decades, few have been reported from the blood that have relevance to the disease [2]. There is thus a lack of robust and reliable blood-based biomarkers for AD diagnosis [3, 4]. With the expanding capacity of protein arrays and mass spectrometry-based detections, recent studies of blood profile biomarkers have attempted to address this problem. Ray and colleagues [5] were the first to use a profiling approach, and they identified an 18-plasma protein profile that classified AD patients from healthy subjects with high specificity. The same group later analyzed independent samples with different bioinformatics approaches and discovered that the majority of those 18 proteins were relevant to the levels of A*β* or tau proteins in CSF [6]. Since these two studies, many profiling approaches have proposed protein panels with promising diagnostic ability, but the main issue has been reproducibility [7]. The problem of reproducibility has been addressed by Hu and colleagues [8] and Doecke and colleagues [9] using two well-characterized and large clinical cohorts to identify a series of inflammatory mediators associated with the onset of AD. Doecke and colleagues [9] and O'Bryant and colleagues [10] also reported high diagnostic accuracy across cohorts. In addition, researchers in plasma proteomics have used cross-validation across various cohorts to overcome the overfitting problem in high-dimensional studies. Molecules that have raised great hopes among these investigators include apolipoprotein E (APOE), NT-proBNP (N-terminal prohormone of Brain Natriuretic Peptide), and pancreatic polypeptide. It is been suggested that, because AD is a mitochondrial dysfunction and immune system relevant disease [11, 12], focusing on genes involved in relevant pathways [13] may help in biomarker discovery [14]. However, few previous studies have used biological information in their modeling. We therefore decided in this study to take existing biological knowledge of potential AD biomarkers into consideration and construct a knowledge feature pool for a series of feature selection methods. We first established a feature pool comprising numerous AD-related biomarkers and then designed two novel SVM-based feature selection methods, which we used to select several panels of biomarkers. Finally, we validated the classifying performance of these panels with other serum and RNA expression cohorts. We found that a panel of only two or three proteins gave us good diagnostic ability.

## 2. Materials and Methods

### 2.1. Data Collection and Preprocessing

We downloaded three AD relevant datasets from Gene Expression Omnibus (http://www.ncbi.nlm.nih.gov/geo/): GSE29676, GSE39087, and GSE5281. GSE29676 consists of serum samples from 50 AD cases, 40 healthy samples, 30 breast cancer (BC) cases, and 29 Parkinson's disease cases. The data were generated by Invitrogen ProtoArray v5.0 protein platform including 9486 unique human protein antigens (dataset feature pool) [15], to which specific proteins will bind when the sample solution is loaded. GSE39087 is also a human serum protein microarray dataset generated by the same platform as GSE29676 and contains 36 AD cases, 57 healthy samples, 48 Parkinson disease cases, 18 breast cancers, and 7 multiple scleroses [16]. GSE5281 is an RNA microarray dataset from brain tissues, with 87 AD cases and 74 healthy samples. Each sample was collected from different brain regions comprising entorhinal cortex (EC), hippocampus (HIP), medial temporal gyrus (MTG), posterior cingulate (PC), superior frontal gyrus (SFG), and primary visual cortex (PVC) [17].

The normalized expression data of GSE29676 and GSE39087 were downloaded directly, then expression values smaller than one were set as one, and 2-based logarithm transformation was conducted. To eliminate the potential bias caused by age and gender, the expression value was corrected using the following method. First, for each protein, a robust linear regression (rlm function in MASS [18] R package) was applied with the logarithm transformed expression value as the dependent variable and age and gender as the explanatory variables. Second, the sum of the intercept and residual was employed as the corrected expression value for that protein in each sample and used in subsequent analyses. For GSE5281, an age-gender-bias correction was also conducted on the normalized data before matching the probes with corresponding proteins. We used GSE29676 as the discovery dataset for biomarker identification and GSE39087 and GSE5281 as the two validation datasets. Only AD and Control subjects were included in any subsequent analysis.

### 2.2. Knowledge-Based Feature Pool

We comprehensively searched the literature and online databases to construct a knowledge feature pool for the AD-related biomarkers. The text mining for AD biomarkers was conducted by searching publications on PubMed in December 2014 (http://www.ncbi.nlm.nih.gov/pubmed), producing a set of 611 genes. 172 genes were discovered from (a) large genome wide association study (GWAS) papers [19–21] and their first neighbors in a protein-protein interaction (PPI) network [22] and (b) AD-related genes and protein database in Alzforum (http://www.alzforum.org/). We collected 84 genes from a human AD real-time PCR array functional gene grouping (http://www.sabiosciences.com/rt_pcr_product/HTML/PAHS-057Z.html) and 876 genes in the Ingenuity Pathway Analysis (http://www.ingenuity.com/) tool filtered by keyword “Alzheimer biomarker.” From these searches a total of 1915 unique genes were placed in our knowledge-based gene pool.

### 2.3. Feature Selection

**Figure 1:**
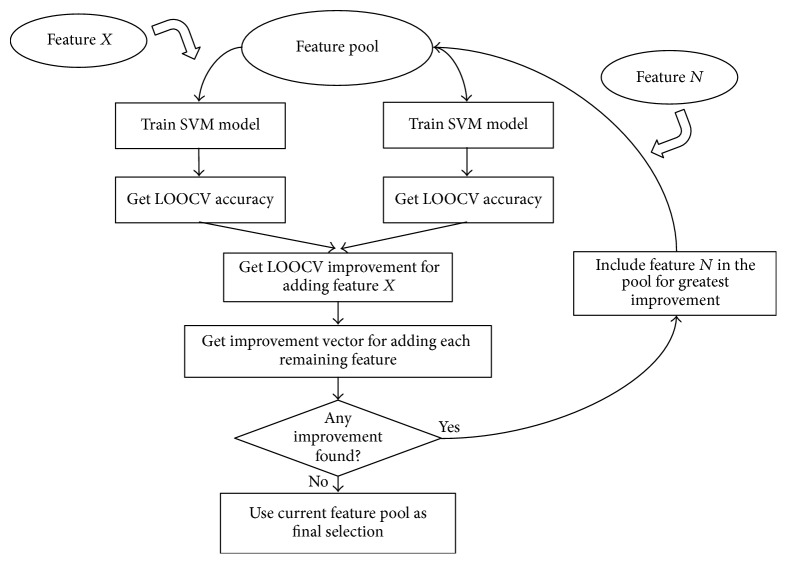
Workflow of Support Vector Machine Forward Selection (SVMFS).

We proposed a novel method, Support Vector Machine Forward Selection (SVMFS), for selecting the best AD-related protein set for training our classification model (Figure 1). The framework of our method is built upon that of the Support Vector Machine (SVM) model. Throughout the study, we adopted the default settings of the SVM model in the e1071 R package [23] (gamma = 1/feature number, cost = 1, type = C-classification, and kernel = radial). For a given protein set, an SVM model can be trained, whose leave-one-out cross-validation (LOOCV) accuracy was then used as the evaluation score. The evaluation score improvement was calculated by comparing the evaluation scores of the previous protein set with the evaluation score of the updated protein set containing a selected additional protein. An alternative feature selection method was also used, SVM Top Forward Selection (SVMTFS). In this alternate method a ranking list for all the proteins based on the LOOCV accuracy of their respective single-protein SVM model was made. The only difference between these two methods lies in the selection of the protein to be included in the next round. In SVMFS, the optimal protein among the rest is selected; in SVMTFS, the next protein in the ranking list based on the LOOCV is selected.

### 2.4. Classifier Training and Assessing

We conducted a cross-validation using the GSE29676 dataset on the protein sets discovered by our novel feature selection approach and the 10 biomarkers discovered by Nagele and colleagues (named here as the Nagele model) [15]. We trained classifiers with 60 samples (randomly selected, 30 each in AD and healthy samples) and then tested the classifiers with the remaining samples (10 AD and 20 healthy samples). The cross-validation was repeated 5000 times for the calculation of average sensitivity, specificity, positive predictive value (PPV), negative predictive value (NPV), false discovery rate (FDR), and false omission rate (FOR) [24]. The ROC curve performance area under the curve (AUC) was plotted using the pROC R package [25].

### 2.5. Biomarker Validation

We conducted both biomarker validation and classification model validation. Classifiers trained in discovery in the GSE29676 dataset were then tested for performance in the GSE39087 dataset. We also did cross-validation using GSE39087 of the features identified by GSE29676, that is, randomly selecting 20 AD and 20 healthy in GSE39087 as training samples and the remainder as testing samples, and repeating 5000 times.

For GSE5281, target proteins identified in discovery dataset were matched with corresponding probes by their corresponding genes. An SVM classification model was built in the six different brain regions separately and LOOCV accuracy was used to assess the performance of the model in each region.

## 3. Results

Employing SVMFS and SVMTFS to select features in the knowledge-based feature pool and full feature pool, respectively, we discovered three different protein sets that showed promising performance in discriminating AD patients from healthy individual as measured by LOOCV accuracy.  Table 1 shows the LOOCV accuracy for each of the top 20 features (proteins) used in a single feature SVM model in discovery dataset. We found the following models (protein sets).

- A two-feature model selected by SVMFS had 98.8% SVM-LOOCV accuracy and consisted of ECH1 + NHLRC2 (enoyl-coenzyme A hydratase 1 peroxisomal plus NHL repeat containing 2).
- A three-feature model selected by SVMFS had 96.5% SVM-LOOCV accuracy and consisted of ERBB2 + FN1 + SLC6A13 (v-erb-b2 erythroblastic leukemia viral oncogene homolog 2, neuro/glioblastoma derived oncogene homolog (avian) transcript variant 2, fibronectin 1, plus solute carrier family 6 (neurotransmitter transporter, GABA), member 13).
- A two-feature model selected by SVMTFS had 97.7% SVM-LOOCV accuracy and consisted of ECH1 + HOXB7 (homeobox B7).

Evaluation by cross-validation in the same dataset showed a good performance of these models (Table 2). The average sensitivity and specificity of models ECH1 + NHLRC2, ECH1 + HOXB7, and ERBB2 + FN1 + SLC6A13 all reached at least 88%. Among the selected proteins, an interesting statistical pattern for the expression level was discovered in ECH1, HOXB7, and ERBB2 (Figures 3 and 4). In each of these three proteins, the normal expression range has two thresholds (one upper limit and one lower limit). To the best of our knowledge, such biomarkers with banded distributions between healthy and AD samples have not previously been reported. Typically there is a binary separation between AD and healthy samples with only one threshold.

### 3.1. Cross-Cohort Validation

**Figure 2:**
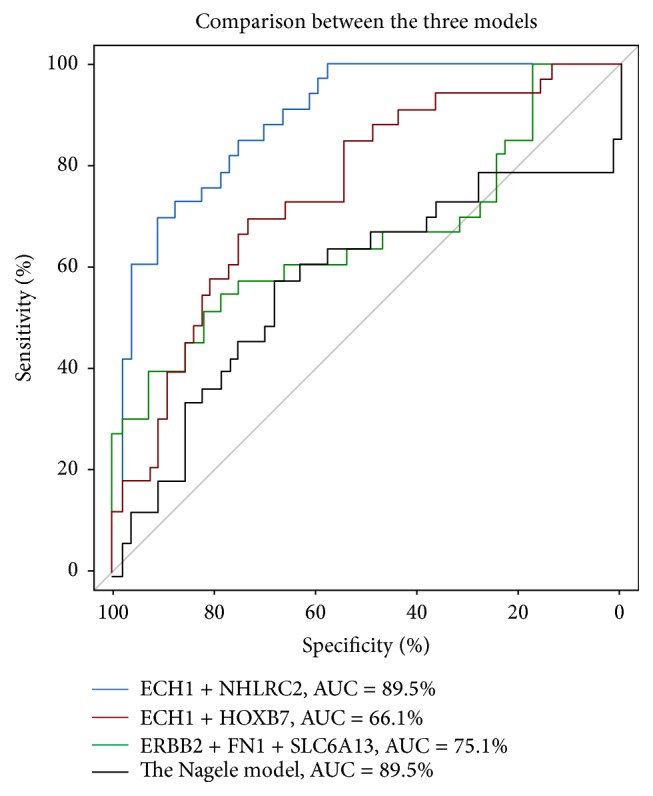
ROC curves of the three proposed models in the cross-cohort validation using GSE39087.

**Figure 3:**
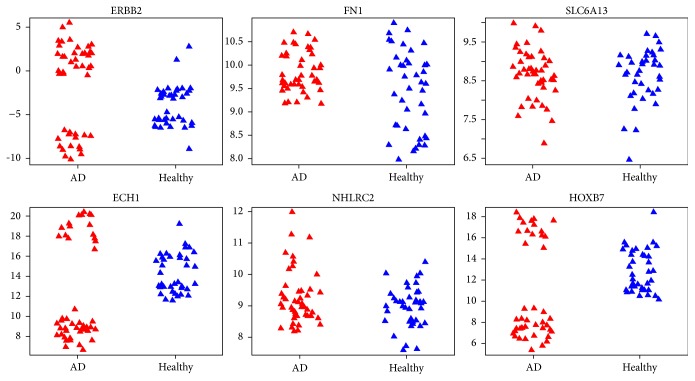
Expression level of six proteins in the three proposed models under different conditions (red for AD samples and blue for healthy samples) in dataset GSE29676. The vertical coordinate of each plot represents the processed expression value and the horizontal coordinate represents different sample categories.

**Figure 4:**
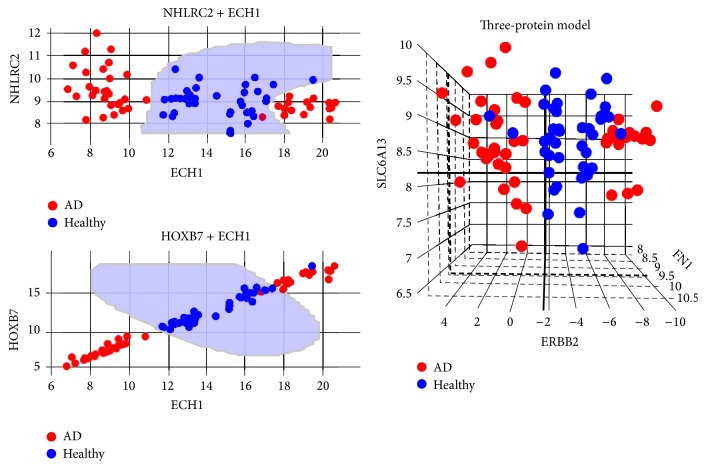
Three proposed models in dataset GSE29676. Blue shaded area indicates where a sample will be classified as healthy by the prediction model. Coordinates in each plot represent the processed expression value. Red represents AD samples and blue represents healthy samples.

The cross-validation using cohort GSE39087, which is also a serum protein microarray data, showed that the three models still maintained good classification ability, with SVM-LOOCV accuracies of 88.9% (ECH1 + NHLRC2), 97.8% (ECH1 + HOXB7), and 74.4% (ERBB2 + FN1 + SLC6A13). Model ECH1 + HOXB7 outperformed the others in this process of validation, with over 95% in sensitivity and specificity (Table 2). Model ECH1 + NHLRC2 also exhibited good predictive performance except for a decreased sensitivity, which could result from the relatively small training sample size. Despite the seemingly good result in cross-validation using GSE39087, the performances of models deteriorated when they were trained and tested by different cohorts (AUC: ECH1 + NHLRC2: 89.5%, ECH1 + HOXB7: 66.1%, ERBB2 + FN1 + SLC6A13: 75.1%; see Figure 2). This could be an indication of overtraining in those models, especially for model ECH1 + HOXB7. The reason for this could be different experimental environments between the two cohorts.

**Figure 5:**
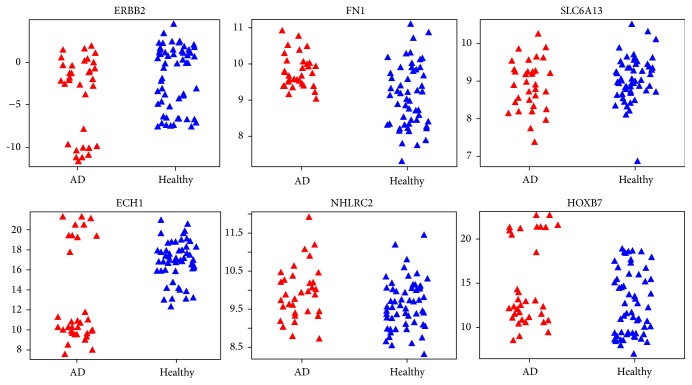
Expression level of six proteins in the three proposed models under different conditions (red for AD samples and blue for healthy samples) in dataset GSE39087.

**Figure 6:**
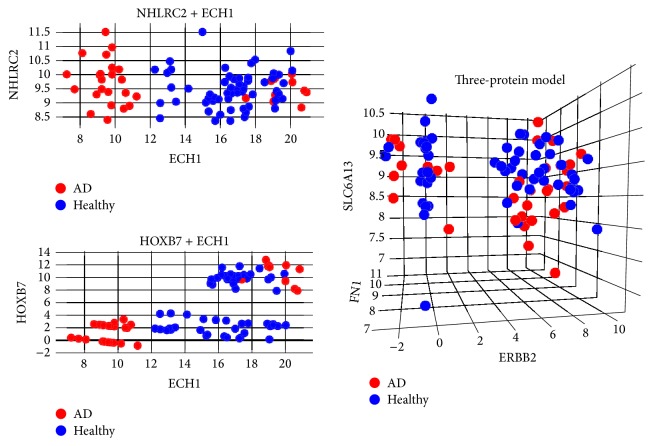
Three proposed models in dataset GSE39087.

We also investigated the distribution pattern for all the proteins using dataset GSE39087 and found that ECH1 still maintained its banded distribution, while in ERBB2 and HOXB7 the patterns are relatively less obvious (Figures 5 and 6). The disparity may be caused by the different data processing methods employed by GSE29676 and GSE39087; the former datasets were characterized into disease and control groups and then linearly normalized while the latter datasets were normalized via the compare-function embedded in Invitrogen's Prospector [26].

We conducted LOOCV separately for our three proposed protein sets in the six different brain regions of dataset GSE5281 (thus, 18 models were evaluated in total). The result shows that our three models maintain excellent classification ability in EC and PC but are poorer in the others (Table 3).

## 4. Discussion

The original study of dataset GSE29676 reported 10 autoantibodies as diagnostic AD biomarkers [15]. The authors constructed a descending ranked list sorted by the difference in prevalence between AD and healthy groups using* Predictive Analysis for Microarrays (PAM)*, and then the top 10 features were selected. This method of feature selection did not take the combinatory effect of feature sets into consideration, as each autoantibody was selected exclusively according to its own discriminant ability between groups. To overcome the weaknesses in feature selection, we used a SVM radial kernel embedded feature selection method, which not only compensates for the ignorance of combinatory effect of significant differentiator feature sets, but also adds the ability to discover complex patterns in the data. More importantly, in the original study the predictive models were trained and validated in samples that were randomly selected, only once, which may lead to uncertain results. In contrast, our study is cross-validated by repeating the sampling for 5000 times to compensate for any uncertainty in bootstrapping. Also, the impacts of age and gender on the prediction models were ignored in Nagele et al.'s study. In a later reexamination by the same researchers, those two factors (age and gender) were identified to strongly influence the number of autoantibodies detected using protein microarrays [16]. We eliminated such effects by simulating a robust linear regression model between age, gender, and the expression value. The expression value was then corrected by summing the intercept and the residue.

We also see a potentially novel pattern of expression in AD and healthy samples with two boundaries. An assumption can be made that there is a normal level of protein expression in healthy individuals. The LOOCV accuracies of those proteins with this particular pattern suggest that any subject with an abnormal expression level, either being up- or downregulated, can be diagnosed as having AD with high confidence. The existence of upper and lower bound of normal expression in these proteins also implies the potential to subdivide AD into two or more categories.

Furthermore, we find a correlation between the expression levels of the proteins with two boundaries in our study. For instance, in the dataset GSE29676, the AD sample group with a downregulated expression level of protein ECH1 compared to normal also have downregulated HOXB7 and upregulated ERBB2 (Pearson correlation *r* = 0.99 for ECH1 and HOXB7; *r* = −0.95 for ECH1 and ERBB2; and *r* = −0.94 for ERBB2 and HOXB7). The same situation was observed in the dataset GSE39087. These observations suggest that there is an underlying linkage between the upstream activities of these proteins. We predict that further investigations will reveal coexpression, regulation, or antagonistic relationship between the precursor molecules of those proteins, at the level of either transcription or translation.

Considering the proteins in our panels, ECH1 is a gene encoding a member of the hydratase/isomerase superfamily. The gene product shows high sequence similarity with the enoyl-coenzyme A (CoA) hydratases of several species, especially within a conserved domain that is characteristic of these proteins. The encoded protein contains a C-terminal peroxisomal targeting sequence that localizes to the peroxisome. Its rat ortholog is a delta3,5-delta2,4-dienoyl-CoA isomerase that functions in the auxiliary step of the fatty acid beta-oxidation pathway. This transcript was reported to be significantly upregulated in response to neuronal silencing in the rat [27] but no linkage to AD or dementia has been reported previously. HOXB7 is a member of the Antp homeobox family and encodes a protein with a homeobox DNA-binding domain. It is included in a cluster of homeobox B genes located on chromosome 17. The encoded nuclear protein functions as a sequence-specific transcription factor that is involved in cell proliferation and differentiation. HOXB7 is age-repressed in mesenchymal stromal cells and conversely age-induced in hematopoietic progenitor cells [28]. ERBB2 is a member of a family of single-transmembrane receptor tyrosine kinases called ERBB and plays the main role in mediating Neuregulin-1 (NRG1) function [29, 30]. NRG1 participates in numerous neurodevelopmental processes and is implicated in nerve cell differentiation and synapse formation [31, 32], radial glia formation and neuronal migration [33, 34], oligodendrocyte development and axon myelination [35, 36], axon navigation [37], and neurite outgrowth [38, 39].

Our findings suggest that the combined expression levels of ECH1, HOXB7, and ERBB2 have good potential to be an indicator of AD pathology. ECH1 and HOXB7 are expressed in almost all tissues and are enriched in the central nervous system, while ERBB2 is absent from many tissues and is not detected in the central nervous system (http://www.proteinatlas.org/). Whether these proteins can pass the blood brain barrier is yet to be investigated.

We note that our approach is different from the recursive feature elimination (RFE) method, which searches features starting from the sorted full feature space and eliminates features by a certain number or proportion in each iteration [15]. In contrast, our approach searches features by including important and informative features in each iteration. Such methods are greedy and may achieve global solutions but are computationally expensive. To overcome this, we restricted our method to include just one feature in each iteration and terminated the searching when the improvement of prediction model caused by including a new feature was less than a predefined threshold (zero in the study).

## 5. Conclusions

The inclusion of existing biological knowledge and use of a novel feature selection method have allowed us to find three protein models that have a promising ability to distinguish AD patients from healthy individuals. We also find a new statistical pattern involving both upper and lower bounds to expression of proteins in our models. The reproducibility of these findings needs now to be tested in larger cohorts.

## Figures and Tables

**Table 1:** Top 20 proteins with the largest LOOCV accuracy.

NCBI accession ID	Protein name	LOOCV accuracy
BC011792.1	ECH1	96.5%
NM_004502.2	HOXB7	96.5%
NM_177924.1	ASAH1	96.5%
BC030814.1	IGKV1-5	95.4%
BC034142.1	IGKV1-5	95.4%
BC034146.1	IGKV1-5	95.4%
BC034937.1	C10orf64	95.4%
NM_176884.1	TAS2R43	95.4%
PV3366	ERBB2	94.2%
NM_201278.1	MTMR2	94.2%
BC038406.1	C3orf20	94.2%
NM_152776.1	MGC40579	94.2%
NM_014110.3	PPP1R8	93.0%
XM_294794.1	LOC339065	93.0%
NM_019891.1	ERO1LB	93.0%
BC068078.1	NPM2	93.0%
NM_002613.3	PDPK1	93.0%
NM_031268.3	PDPK1	93.0%
BC032101.1	JAGN1	93.0%
NM_000963.1	PTGS2	93.0%

**Table 2:** Performances of three proposed models in datasets GSE29676 and GSE39087.

	Average LOOCV accuracy	Validation accuracy	Sensitivity	Specificity	NPV	PPV	FDR	FOR
Cross-validation in GSE29676								
ECH1 + NHLRC2	98.8%	95.4%	94.0%	97.1%	93.0%	97.7%	2.3%	7.0%
ECH1 + HOXB7	97.7%	95.6%	95.0%	96.3%	94.1%	97.1%	2.9%	5.9%
ERBB2 + FN1 + SLC6A13	96.5%	89.6%	87.9%	91.8%	86.4%	93.4%	6.6%	13.6%
The Nagele model	64.0%	56.8%	58.9%	54.3%	51.8%	62.2%	37.8%	48.2%

Cross-validation in GSE39087								
ECH1 + NHLRC2	88.9%	87.4%	79.7%	90.8%	91.2%	80.7%	19.4%	8.8%
ECH1 + HOXB7	97.8%	96.9%	96.8%	96.9%	98.6%	93.8%	6.2%	1.4%
ERBB2 + FN1 + SLC6A13	74.4%	69.8%	80.9%	65.0%	89.4%	51.4%	48.6%	10.6%
The Nagele model	70.0%	69.4%	80.2%	64.7%	89.0%	50.6%	49.4%	11.0%

**Table 3:** Accuracy performances of our three proposed models in dataset GSE5281 (see Section 2 for full name of brain regions).

	EC	HIP	MTG	PC	SFG	VCX
ECH1 + NHLRC2	95.5%	78.3%	60.0%	57.1%	68.0%	50.0%
ECH1 + HOXB7	86.4%	87.0%	80.0%	85.7%	68.0%	40.0%
ERBB2 + FN1 + SLC6A13	90.9%	56.5%	88.0%	81.0%	60.0%	43.3%

## References

[1] Prince R., Wimo A., Guerchet M., Ali G. C., Wu Y., Prina M. (2015). *World Alzheimer Report 2015*.

[2] Schreitmüller B., Leyhe T., Stransky E., Köhler N., Laske C. (2012). Elevated angiopoietin-1 serum levels in patients with alzheimer's disease. *International Journal of Alzheimer's Disease*.

[3] Hampel H., Lista S., Teipel S. J. (2014). Perspective on future role of biological markers in clinical therapy trials of Alzheimer's disease: a long-range point of view beyond 2020. *Biochemical Pharmacology*.

[4] Patel S., Shah R. J., Coleman P., Sabbagh M. (2011). Potential peripheral biomarkers for the diagnosis of Alzheimer's disease. *International Journal of Alzheimer's Disease*.

[5] Ray S., Britschgi M., Herbert C. (2007). Classification and prediction of clinical Alzheimer's diagnosis based on plasma signaling proteins. *Nature Medicine*.

[6] Britschgi M., Rufibach K., Huang S. L. B. (2011). Modeling of pathological traits in Alzheimer's disease based on systemic extracellular signaling proteome. *Molecular & Cellular Proteomics*.

[7] Björkqvist M., Ohlsson M., Minthon L., Hansson O. (2012). Evaluation of a previously suggested plasma biomarker panel to identify Alzheimer's disease. *PLoS ONE*.

[8] Hu W. T., Holtzman D. M., Fagan A. M. (2012). Plasma multianalyte profiling in mild cognitive impairment and Alzheimer Disease. *Neurology*.

[9] Doecke J. D., Laws S. M., Faux N. G. (2012). Blood-based protein biomarkers for diagnosis of Alzheimer disease. *Archives of Neurology*.

[10] O'Bryant S. E., Xiao G., Barber R. (2011). A blood-based screening tool for Alzheimer's disease that spans serum and plasma: findings from TARC and ADNI. *PLoS ONE*.

[11] Moreira P. I., Carvalho C., Zhu X., Smith M. A., Perry G. (2010). Mitochondrial dysfunction is a trigger of Alzheimer's disease pathophysiology. *Biochimica et Biophysica Acta-Molecular Basis of Disease*.

[12] Lambert J.-C., Grenier-Boley B., Chouraki V. (2010). Implication of the immune system in Alzheimer's disease: evidence from genome-wide pathway analysis. *Journal of Alzheimer's Disease*.

[13] Mattson M. P. (2004). Pathways towards and away from Alzheimer's disease. *Nature*.

[14] Ruffner H., Bauer A., Bouwmeester T. (2007). Human protein-protein interaction networks and the value for drug discovery. *Drug Discovery Today*.

[15] Nagele E., Han M., DeMarshall C., Belinka B., Nagele R. (2011). Diagnosis of Alzheimer's disease based on disease-specific autoantibody profiles in human sera. *PLoS ONE*.

[16] Nagele E. P., Han M., Acharya N. K., DeMarshall C., Kosciuk M. C., Nagele R. G. (2013). Natural IgG autoantibodies are abundant and ubiquitous in human sera, and their number is influenced by age, gender, and disease. *PLoS ONE*.

[17] Liang W. S., Reiman E. M., Valla J. (2008). Alzheimer's disease is associated with reduced expression of energy metabolism genes in posterior cingulate neurons. *Proceedings of the National Academy of Sciences of the United States of America*.

[18] Venables W. N., Ripley B. D. (2002). *Modern Applied Statistics with S*.

[19] Cruts M., Theuns J., Van Broeckhoven C. (2012). Locus-specific mutation databases for neurodegenerative brain diseases. *Human Mutation*.

[20] Strittmatter W. J., Saunders A. M., Schmechel D. (1993). Apolipoprotein E: high-avidity binding to *β*-amyloid and increased frequency of type 4 allele in late-onset familial Alzheimer disease. *Proceedings of the National Academy of Sciences of the United States of America*.

[21] Williams J., Amouyel P. (2013). Meta-analysis of 74,046 individuals identifies 11 new susceptibility loci for Alzheimer's disease. *Nature Genetics*.

[22] Li X., Long J., He T., Belshaw R., Scott J. (2015). Integrated genomic approaches identify major pathways and upstream regulators in late onset Alzheimer's disease. *Scientific Reports*.

[23] Dimitriadou E., Hornik K., Leisch F., Meyer D., Weingessel A. (2005). Misc functions of the department of statistics (e1071) TU Wien.

[24] Fletcher H., Suzanne W. (2005). *Clinical Epidemiology: The Essentials*.

[25] Robin X., Turck N., Hainard A. (2011). pROC: an open-source package for R and S+ to analyze and compare ROC curves. *BMC Bioinformatics*.

[26] Thermo Fisher Scientific (2015). *ProtoArray Prospector v5.2.3*.

[27] Gleichmann M., Zhang Y. Q., Wood W. H. (2012). Molecular changes in brain aging and Alzheimer's disease are mirrored in experimentally silenced cortical neuron networks. *Neurobiology of Aging*.

[28] Wagner W., Bork S., Horn P. (2009). Aging and replicative senescence have related effects on human stem and progenitor cells. *PLoS ONE*.

[29] Yarden Y., Sliwkowski M. X. (2001). Untangling the ErbB signalling network. *Nature Reviews Molecular Cell Biology*.

[30] Falls D. L. (2003). Neuregulins: functions, forms, and signaling strategies. *Experimental Cell Research*.

[31] Buonanno A., Fischbach G. D. (2001). Neuregulin and ErbB receptor signaling pathways in the nervous system. *Current Opinion in Neurobiology*.

[32] Corfas G., Roy K., Buxbaum J. D. (2004). Neuregulin 1-erbB signaling and the molecular/cellular basis of schizophrenia. *Nature Neuroscience*.

[33] Anton E. S., Marchionni M. A., Lee K.-F., Rakic P. (1997). Role of GGF/neuregulin signaling in interactions between migrating neurons and radial glia in the developing cerebral cortex. *Development*.

[34] Rio C., Rieff H. I., Qi P., Corfas G. (1997). Neuregulin and erbB receptors play a critical role in neuronal migration. *Neuron*.

[35] Fernandez P.-A., Tang D. G., Cheng L., Prochiantz A., Mudge A. W., Raff M. C. (2000). Evidence that axon-derived neuregulin promotes oligodendrocyte survival in the developing rat optic nerve. *Neuron*.

[36] Calaora V., Register B., Bismuth K. (2001). Neuregulin signaling regulates neural precursor growth and the generation of oligodendrocytes in vitro. *Journal of Neuroscience*.

[37] López-Bendito G., Cautinat A., Sánchez J. A. (2006). Tangential neuronal migration controls axon guidance: a role for neuregulin-1 in thalamocortical axon navigation. *Cell*.

[38] Bermingham-McDonogh O., McCabe K. L., Reh T. A. (1996). Effects of GGF/neuregulins on neuronal survival and neurite outgrowth correlate with erbB2/neu expression in developing rat retina. *Development*.

[39] Gerecke K. M., Wyss J. M., Carroll S. L. (2004). Neuregulin-1*β* induces neurite extension and arborization in cultured hippocampal neurons. *Molecular and Cellular Neuroscience*.

